# Adjuvant Effects of a CC Chemokine for Enhancing the Efficacy of an Inactivated *Streptococcus agalactiae* Vaccine in Nile Tilapia (*Oreochromis niloticus*)

**DOI:** 10.3390/vaccines12060641

**Published:** 2024-06-08

**Authors:** Chayanit Soontara, Anurak Uchuwittayakul, Pattanapon Kayansamruaj, Piti Amparyup, Ratree Wongpanya, Prapansak Srisapoome

**Affiliations:** 1 Laboratory of Aquatic Animal Health Management, Department of Aquaculture, Faculty of Fisheries, Kasetsart University, 50 Paholayothin Rd., Ladyao, Chatuchak, Bangkok 10900, Thailand; chayanit.soo@ku.th (C.S.); ffisarb@ku.ac.th (A.U.); pattanapon.k@ku.ac.th (P.K.); 2 Center of Excellence in Aquatic Animal Health Management, Faculty of Fisheries, Kasetsart University, 50 Paholayothin Rd., Ladyao, Chatuchak, Bangkok 10900, Thailand; 3 Marine Biotechnology Research Team, Integrative Aquaculture Biotechnology Research Group, National Center for Genetic Engineering and Biotechnology (BIOTEC), National Science and Technology Development Agency (NSTDA), 113 Paholyothin Road, Klong 1, Khlong Luang 12120, Thailand; piti.amp@biotec.or.th; 4 Department of Biochemistry, Faculty of Science, Kasetsart University, 50 Ngamwongwan Road, Bangkok 10900, Thailand; fscirtw@ku.ac.th

**Keywords:** Nile tilapia, immune response, CC chemokine, *Streptococcus agalactiae*, adjuvant, vaccine, transcriptome, immune-related gene

## Abstract

In this study, the ability of a CC chemokine (*On*-CC1) adjuvant to enhance the efficacy of a formalin-killed *Streptococcus agalactiae* vaccine (WC) in inducing immune responses against *S. agalactiae* in Nile tilapia was investigated through immune-related gene expression analysis, enzyme-linked immunosorbent assay (ELISA), transcriptome sequencing, and challenge tests. Significantly higher *S. agalactiae*-specific IgM levels were detected in fish in the WC+CC group than in the WC alone or control groups at 8 days postvaccination (dpv). The WC vaccine group exhibited increased specific IgM levels at 15 dpv, comparable to those of the WC+CC group, with sustained higher levels observed in the latter group at 29 dpv and after challenge with *S. agalactiae* for 14 days. Immune-related gene expression analysis revealed upregulation of all target genes in the control group compared to those in the vaccinated groups, with notable differences between the WC and WC+CC groups at various time intervals. Additionally, transcriptome analysis revealed differential gene expression profiles between the vaccinated (24 and 96 hpv) and control groups, with notable upregulation of immune-related genes in the vaccinated fish. Differential gene expression (DGE) analysis revealed significant upregulation of immunoglobulin and other immune-related genes in the control group compared to those in the vaccinated groups (24 and 96 hpv), with distinct patterns observed between the WC and WC+CC vaccine groups. Finally, challenge with a virulent strain of *S. agalactiae* resulted in significantly higher survival rates for fish in the WC and WC+CC groups compared to fish in the control group, with a notable increase in survival observed in fish in the WC+CC group.

## 1. Introduction

Nile tilapia (*Oreochromis niloticus*) is a major component of the freshwater fishery industry of Thailand and has a substantial impact on the economy via its contribution to profit and support for various industries, both domestically and globally. As reported by the Department of Fisheries of Thailand, the overall production from freshwater aquaculture in the country reached 466,953 tons in 2022. Notably, Nile tilapia constitute a significant portion of that production, contributing 57.7%, or 269,400 tons, to the total production, with an estimated value of THB 12,613 million [1].

However, the tilapia farming industry is currently facing a serious challenge due to recurring diseases linked to intensive cultivation practices and fluctuating environmental conditions. *Streptococcus agalactiae* is the predominant bacterial disease, and both serotypes Ia and III have been found to cause widespread outbreaks and tilapia mortality in Thailand, resulting in significant and persistent damage induced by infectious diseases [2]. To address this, farmers have employed chemicals and antibiotics, but there are currently concerns about the potential development of antibiotic-resistant pathogenic bacteria and the remaining parts of these compounds in the environment and consumers [3,4,5,6].

Consequently, there is a growing recognition of the need for sustainable solutions to address challenges within the tilapia aquaculture industry in Thailand. One approach involves enhancing disease resistance to promote animal health. Vaccination, a widely accepted and safe method, includes the administration of inactivated vaccines through injection [7,8,9]. However, some strains induce suboptimal responses, hindering the elicitation of a sufficient immune response. An adjuvant can enhance disease prevention when used alongside vaccination by improving antigen processing, presentation, and recognition [10,11,12]. Ideal adjuvant characteristics include increased vaccine effectiveness for prophylaxis, enhanced immunogenicity, and minimal-to-no side effects in animals [10,11,12]. However, options for adjuvants in the fish aquaculture industry, including for Nile tilapia, are currently limited.

CC chemokines, small proteins crucial for immune responses and inflammation, play a crucial role in directing leukocyte chemotaxis and activation [13,14]. They are also crucially involved in immune cell trafficking and regulate movement within tissues during various pathological and physiological processes, including inflammation and immune surveillance [13,14]. In our previous study, two CC chemokine proteins, *On*-CC1 and *On*-CC2, were identified in Nile tilapia [15]. These two recombinant *On*-CCs demonstrated rapid immune system stimulation within 6 h, enhancing tilapia leukocyte phagocytosis [15], indicating their potential for application as vaccine adjuvants.

Based on the current information, chemokines have recently been explored as potent adjuvants for vaccination in various teleosts, although not in Nile tilapia. Chemokines have been investigated for their ability to bolster protective immunity and their capacity to regulate immune responses. Chemokine adjuvants can influence the polarization and intensity of immune reactions induced by peptide, protein, subunit, or DNA vaccines [16,17,18,19,20]. As naturally occurring products of the body’s immune system, chemokines present the potential for safer and more dependable adjuvant activity than synthetic alternatives [21]. Therefore, using these immunostimulators could enhance vaccine safety, efficacy, and versatility, providing nontoxic, well-understood mechanisms that can be adapted to different vaccine types and administration modes.

Thus, the objective of the present research was to evaluate the effectiveness of the *On*-CC1 chemokine protein as an adjuvant for enhancing immune defense mechanisms and disease resistance against pathogenic *S. agalactiae* infection in Nile tilapia. Transcriptome analysis is a powerful tool for describing gene expression responses under various conditions at the molecular level [22,23,24,25], including vaccination-induced responses in various fish species [26,27,28]. Similarly, transcriptome analysis of the head kidney of control and vaccinated fish clearly revealed the upregulation of vital immune-related genes induced by a whole-cell inactivated vaccine (WC) and enhanced induction by the WC *On*-CC1 adjuvant plus (WC+CC) vaccine. The information obtained from this study provides a crucial understanding of the biological immune functions of the *On*-CC1 chemokine in association with the immune system of Nile tilapia and further demonstrates its efficacy in improving streptococcosis disease resistance as an effective adjuvant during vaccination with whole-cell-based inactivated vaccines. This information is vital for vaccine development to improve Nile tilapia production by preventing harmful diseases caused by various severely pathogenic bacteria, with the potential for a crucial impact on the eco-friendly sustainability of Nile tilapia aquaculture through its potential as an alternative to the application of harmful chemicals and drugs.

## 2. Materials and Methods

### 2.1. Experimental Animals

Five hundred fingerlings of Nile tilapia with an average weight of 30.0 ± 3.75 g were acclimatized for seven days in a 1000 L polyethylene tank (100 fish per tank) with full aeration at a temperature of 28 ± 2 °C, a pH of 7.85 ± 1, and a 12-h light: 12-h dark photoperiod. The fish were fed commercial pelleted feed (CPF, Samut Prakan, Thailand) at 5% of their body weight per day. Fifty percent of the water was replaced every three days. Prior to conducting the experiment, the internal organs (liver, spleen, and head kidney) of five randomly selected tilapia were examined for the presence of tilapia lake virus (TiLV) and bacterial pathogens [29]. PCR and microbiological techniques were used to confirm the health status of the fish.

The experimental protocols involving animal utilization for scientific purposes were carried out according to the Ethical Principles and Guidelines for the Use of Animals (National Research Council of Thailand). The procedures were approved by the Animal Ethics Committee of Kasetsart University, Thailand (ethics ID: ACKU63-FIS-006, 10 August 2020).

### 2.2. Preparation of an Inactivated Streptococcus agalactiae Vaccine

The *S. agalactiae* serotype III strain AQSA001 was initially isolated from infected Nile tilapia in Thailand and further identified by the Laboratory of Aquatic Animal Health Management (LAB AAHM), Department of Aquaculture, Faculty of Fisheries, Kasetsart University, Thailand. The target bacterium was grown on brain heart infusion agar (BHIA, HiMedia, Maharashtra, India) at 32 °C for 18–24 h. Subsequently, a single colony of pure *S. agalactiae* was resuspended in 100 mL of BHI broth (BHIB, HiMedia, India) and shaken at 32 °C for 18–24 h. The bacterial suspension was washed with 0.85% sterile NaCl by centrifugation at 2500× *g* (Hettich^®^ Universal 320R, Burladingen, Germany) for 15 min, and the concentration of bacteria was adjusted spectrophotometrically to an absorbance of 0.669 at an optical density (OD) of 600 nm (equivalent to 1 × 10^9^ CFU/mL). Viable cells of the prepared bacteria were killed by exposing 100 mL of the bacterial solution to 1 mL of formalin solution (37% formaldehyde, Sigma–Aldrich, MO, USA) at a final concentration of 1% formalin. The inactivated bacteria were kept at 4 °C for 24 h, washed once with sterile NaCl, and resuspended in sterile NaCl at a target concentration of 1 × 10^9^ CFU/mL. To confirm inactivation, the inactivated bacteria were carefully streaked onto BHI agar and finally incubated at 32 °C for 24 h.

### 2.3. Production of Recombinant rOn-CC1 Protein

cDNA encoding the mature protein for the *On*-CC1 gene was constructed using the pET-28b(+) expression vector (Thermo Fisher Scientific, Waltham, MA, USA) as previously described [15]. Briefly, the resulting plasmids were transformed into the *Escherichia coli* strain BL21 using methods described in a previous study [15]. The recombinant plasmids were extracted from the positive selected clones using a Plasmid Extraction Mini Kit (Thermo Fisher Scientific, Waltham, MA, USA) following the manufacturer’s instructions. The positivity of the selected clones was confirmed through restriction enzyme digestion using plasmid DNA from PCR-screened colonies, as previously described. Uncut plasmid DNA was further validated by DNA sequencing to ensure accurate insertion of the corrected nucleotide sequences, as previously described [15]. A positive colony of an r*On*-CC1 clone was added to 10 mL of Luria–Bertani (LB) broth supplemented with kanamycin (30 μg/mL) and grown in an orbital shaker at 37 °C for 24 h. Then, 400 μL of the obtained bacterial suspension was transferred to 400 mL of LB broth supplemented with a similar concentration of the previous kanamycin solution and incubated at 37 °C in a shaking incubator at 160 rpm until the absorbance reached 0.6 at OD600.

The expression of r*On*-CC1 was carefully stimulated by supplementing isopropyl-β-D-thiogalactoside (IPTG) (Merck, Darmstadt, Germany) to achieve a target concentration of 1 mM IPTG in the culture medium, followed by shaking at 37 °C for 3 h. Viable bacterial cells were collected by centrifugation at 2500× *g* and lysed in 8 mL of guanidinium lysis buffer (20 mM sodium phosphate, 6 M guanidine hydrochloride, and 500 mM NaCl, pH 7.8). The bacterial lysate solution was then sonicated for 7 s (five cycles) and centrifuged at 15,000× *g* at 4 °C for 5 min. The induced r*On*-CC1 was purified using a Ni-NTA Purification System (Invitrogen^TM^, USA) under specific hybrid conditions, a method employed to maintain the function of insoluble proteins using 15 mL of solubilization buffer composed of 20 mM Tris-HCl, 0.5 M NaCl, 5 mM imidazole, 6 M urea, and 1 mM 2-mercaptoethanol (pH 8.0).

Following the protein purification process, the hybrid protein was carefully eluted with elution buffer (pH 8.0), which contained 3 M imidazole and 1× native purification buffer (pH 6.0). The resulting r*On*-CC1 fraction was collected using a 1.5 mL microcentrifuge tube, and the absorbance was measured at OD 280 nm. The fractions with the highest absorbances were then combined, and dialysis at pH 8.0 (0.1% Triton X-100 and 10 mM Tris) was conducted overnight at 4 °C to remove other small molecules such as salts and urea.

Ten microliters of purified r*On*-CC1 was subjected to SDS–PAGE analysis following previously described procedures. The remaining purified protein was then stored at −80 °C until needed. Next, a Coomassie (Bradford) Protein Assay Kit (Thermo Scientific™, MA, USA) was used to quantify the total concentration of protein at an OD of 595 nm using a Bio-Rad microplate reader (Bio-Rad, CA, USA). The concentration of protein was measured using absorbance values calibrated with standard protein concentrations according to the manufacturer’s instructions.

### 2.4. Application of CC Chemokine Adjuvants to Enhance the Efficacy of Inactivated S. agalactiae Vaccines

#### 2.4.1. Experimental Design and Vaccination

Pathogen-free Nile tilapia, described in Section 2.1 above, were separated into four different groups. The fish in all groups were kept in 250 L tanks at a stocking density of 30 fish/tank, and 4 replications (4 tanks/group) were performed for each group. The fish were acclimatized to the conditions described above for 1 week. The fish in the control group were injected via the intraperitoneal (IP) route with 0.1 mL of phosphate-buffered saline (PBS, pH 7.4), while the fish in the second and third groups were also IP injected with 0.1 mL of the inactivated *S. agalactiae* vaccine (WC) or 1 × 10^6^ CFU of formalin-killed *S. agalactiae* containing 10 µg of r*On*-CC1 (WC+CC). During this period, growth parameters were investigated using protocols described previously [29] at 29 days postvaccination (dpv), one day before the challenge test.

#### 2.4.2. Sample Collection

Serum and immune-related organs, including the head kidney and spleen, were collected at 0, 1, 8, 15, 22, and 29 dpv from one fish in each replicate (*n* = 4) using previously described methods [30]. The serum was kept at −20 °C for later use in indirect enzyme-linked immunosorbent assays (ELISAs) and immunological activity assays, while the internal organs were placed in PE tubes containing 10% neutral buffered formalin preservative for subsequent histological processing.

To analyze immune gene expression, spleen and head kidney samples were collected at 0, 6, 24, 72, and 168 hpv from one fish in each replicate.

For transcriptomic analysis, head kidneys from fishes in the WC- and WC+CC-vaccinated groups were collected at 24 and 96 hpv from one fish in each replicate (*n* = 4/group). For the control, transcriptome analysis of the head kidney samples was performed only at 96 hpv after injection with PBS, and the results were then compared with those of both the 24 and 96 hpv samples from the WC- and WC+CC-vaccinated groups. The tissues were placed in 1.5 mL Eppendorf tubes containing 1.0 mL of NucleoZol (Macherey-Nagel, Dueren, Germany) at −20 °C for subsequent RNA extraction and transcriptomic sequencing. The remaining fish (20 fish per tank) were then subjected to an experimental challenge to assess resistance to *S. agalactiae* infection.

#### 2.4.3. Assessment of the Levels of Serum IgM Antibodies Specific to *S. agalactiae* Using ELISA

An indirect ELISA was conducted to quantify serum IgM levels specific to *S. agalactiae* following a previous procedure, with some modifications [30]. Briefly, the inactivated formalin-killed *S. agalactiae* antigen was washed with PBS and resuspended in coating buffer (carbonate-bicarbonate solution, pH 9.6) to a final concentration of 1 × 10^8^ CFU/mL. A total volume of 0.1 mL of antigen solution was added to each well of a MaxiSorp™ flat-bottom 96-well plate with surface treatment, high affinity to molecules, mixed with hydrophilic/hydrophobic domains (Thermo Fisher Scientific, CA, UA), and incubated overnight at 4 °C. The coated plate was gradually washed thrice with Tris-buffered saline-tween (TBST) wash buffer containing 0.05% Tween-20 (0.02 M Tris; 0.38 M NaCl; 0.05% Tween 20, pH 7.3). Blocking buffer containing 1% *w*/*v* bovine serum albumin was added to the plate to block nonspecific binding sites, and the plates were incubated for 2 h at room temperature (RT). Afterward, the plates were washed three times with TBST wash buffer. One hundred microliters of fish serum (1:100 dilution in PBS) was added and incubated for 3 h at RT, followed by washing 3 times with TBST wash buffer. Tkj fcx hen, a 1:200 dilution of mouse anti-tilapia IgM (Aquatic Diagnostics Ltd., Stirling, UK) was added at 0.1 mL/well, and the mixture was incubated for 60 min at RT before being washed three times with TBST. Conjugated anti-mouse IgG-HRP was diluted 1:1000, and 0.1 mL of the diluted secondary antibody was added to each well, followed by washing six times with TBST. Finally, 0.1 mL of TMB substrate (Biofx^®^, MD, USA) was added and incubated for 10 min at RT. To stop the reaction, 0.1 mL of 2 M H_2_SO_4_ was added and absorbance at OD450 nm was recorded using a microplate reader (Bio-Rad 680 microplate reader, Hercules, CA, USA). The wells that did not contain fish serum were considered to have a negative cutoff value.

#### 2.4.4. Analysis of Immune-Related Gene Expression

##### Total RNA Extraction and First-Strand cDNA Synthesis

Total RNA was extracted from the head kidney and spleen of the fish using NucleoZOL reagent (Macherey-Nagel, Dueren, Germany) according to the manufacturer’s protocol. Approximately 50 mg of tissue was homogenized in 500 μL of NucleoZOL (Macherey-Nagel, Germany). Subsequently, RNA quantification was performed using a NanoDrop 2000C spectrophotometer (Thermo Fisher Scientific, Waltham, MA, USA). The RNA was diluted to 500 ng/μL with RNase-free water. Total RNA (1000 ng) was subjected to first-strand cDNA synthesis using ReverTra Ace^TM^ qPCR RT Master Mix supplemented with gDNA Remover (Toyobo, Satte City, Japan) following the manufacturer’s instructions. The obtained cDNA was stored at −20 °C.

##### Quantitative Real-Time PCR (qRT–PCR) Analysis

The relative expression levels of eight immune-related genes, *IgM*, *On*-*CC1*, *On*-*CC2TNF-α*, *IFN-γ*, *IL-1β*, *IL-8(1)* (*CXC1*), and *IL*-*8(2)* (*CXC2*), in the spleen and head kidney upon vaccination were determined with the specific primers displayed in Table 1. The reaction mixture contained 500 ng of cDNA, 5 μL of 1X master mix (Hi-Chrom PCR Master mix, HiMedia^®^, India), 150 nM of each primer; pair, and DNase-free water up to a final volume of 10 µL. The real-time PCR conditions included an initial denaturation step of 3 min at 95 °C, followed by 40 cycles of 10 s at 95 °C, 30 s at 60 °C, and 30 s at 72 °C, with a final step of 5 min at 72 °C.

The *β-actin* gene served as a reference gene to normalize the gene expression levels. The 2^−ΔΔCT^ method was used to calculate the relative expression ratio of the target immune-related genes in Nile tilapia tissues at various time intervals [31].

**Table 1:** Primers for investigating immune-related gene expression using qRT–PCR.

Gene	Sequence (5′ to 3′)		Amplicon Size (bp)	References
β-actin	F	ACAGGATGCAGAAGGAGATCACAG	155	[32]
	R	GTACTCCTGCTTGCTGATCCACAT		
Immunoglobulin M (*IgM*)	F	AGGCACAACGGTCACTGTCA	108	[32]
	R	GCAAGGCAGCCAAGAGTGAC		
Interferon gamma (*IFN-γ*)	F	CAGCAGAGATGAACTTGA	93	[32]
	R	CACTAGGAAATACGGGTTT		
Interleukin-1β (*IL-1β*)	F	GTGCTGAGCACAGAATTCCAGGAT	166	[32]
	R	GAAGAACCAAGCTCCTCTTTTGGC		
Tumor necrosis factor alpha (*TNF-α*)	F	CTGTAGTCACCTCCATTA	94	[32]
	R	TACTTGTTGTTGCTTCTG		
Interleukin-8 (1) (*IL-8(1) or CXC1*)	F	TGTCTGTGTCACCGTGTCAGGAAT	151	[32]
	R	CCTTCAGCTCAGGGTTCAAGCAAT		
Interleukin-8 (2) (*IL-8(2) or CXC2*)	F	CAAGCAGGACAACAGTGTCTGTGT	102	[32]
	R	GTTGCAGAATTTGGTTGCTGGGTAG		
CC chemokine1 (*On-CC1*)	F	ACAGAGCCGATCTTGGGTTACTTG	228	[32]
	R	TGAAGGAGAGGCGGTGGATGTTAT		
CC chemokine2 (*On-CC2*)	F	TGGGTTCGTGCCAAGATTGTTGCA	120	[32]
	R	TGAAGGAGAGGCGGTGGATGTTAT		

#### 2.4.5. Challenge Test

To prepare viable bacteria for the challenge assay, a culture of *S. agalactiae* AQSA001 was incubated in 100 mL of TSB medium in an orbital shaker at 32 °C for 18 h. Viable bacterial cells were then centrifuged at 2500 rpm for 10 min. The obtained bacterial cells were further washed and finally dissolved in PBS at pH 7.4. The concentration was 1 × 10^8^ CFU/mL. Following a 30-day vaccination period, 20 fish from each replicate (*n* = 80/group) were intraperitoneally injected with 0.1 mL of the bacterial solution, based on preliminary median lethal dose (LD_50_) data from a previous study [29]. The challenge dose was 1 × 10^7^ CFU/fish. The mortality of the injected fish was recorded daily for 14 days, and confirmation of the disease in moribund fish involved isolation on TSA to verify the presence of the *S. agalactiae* bacterial pathogen. Relative percent survival (RPS) was also investigated with a previously reported method [29].

#### 2.4.6. Statistical Analysis

All obtained results are shown as the mean ± standard deviation (SD). One-way analysis of variance (ANOVA) was used for statistical analysis. The levels of significant differences between the experimental groups and the control group for all observed parameters are indicated by * (*p* < 0.05) and ** (*p* < 0.01). Duncan’s new multiple range test (DMRT) was used to compare significant differences between the tested groups. The cumulative survival of the Nile tilapia challenged with bacteria was calculated using Kaplan–Meier survival curve analysis. All the statistical analyses were performed with the Statistical Package for Social Science (SPSS for Mac version 24.0 Chicago, IL, USA).

### 2.5. Transcriptome Analysis

#### 2.5.1. RNA Isolation, cDNA Library Preparation, and Sequencing Analysis

Total RNA was isolated from the head kidney of fish in each group at two time points, 24 and 96 hpv, following the protocol described above. The quality and concentration of RNA were assessed using a NanoDrop spectrophotometer (Thermo Scientific^TM^, CA, USA) and agarose gel electrophoresis. Subsequently, the RNA-seq library was constructed and sequenced on an Illumina HiSeq 4000 sequencer at Novogene Co., Ltd. (Cambridge, UK).

#### 2.5.2. Transcriptomic Bioinformatics

The RNA-seq data for each cDNA library were annotated on the basis of the assembled unigenes within relevant databases, including the NCBI database (https://www.ncbi.nlm.nih.gov/, accessed on 9 November 2023) accessed through the BlastN search program. Moreover, for nonredundant (NR) protein database searches, we utilized BlastX, which includes resources such as the eukaryotic orthologous groups of proteins (KOGs) (https://mycocosm.jgi.doe.gov/help/kogbrowser.jsf, accessed on 9 November 2023), Kyoto Encyclopedia of Genes and Genome (KO or KEGG Orthology) (https://www.genome.jp/kegg/, accessed on 2 December 2023), Gene Ontology (GO) (http://geneontology.org/, accessed on 2 December 2023), Pfam or InterPro (Pfam) (https://www.ebi.ac.uk/interpro/, accessed on 2 December 2023), and Swiss-Prot (Swissport) (https://www.uniprot.org/, accessed on 2 December 2023) databases.

#### 2.5.3. Analysis of Differentially Expressed Genes (DEGs)

The initial identification of candidate genes was based on their expression levels via fragments per kilobase of transcript per million mapped reads (FPKM) analysis. DESeq software v2 was utilized for biological replicates, with significance thresholds set at *p* < 0.05. Subsequently, KEGG enrichment and GO analyses were conducted using KOBAS v2.0. and 12GOSeq v2.1, respectively.

## 3. Results

### 3.1. Production of Recombinant On-CC1 (rOn-CC1) and Growth Parameters after Vaccination

In this study, r*On*-CC1 was successfully expressed, and high concentrations of the protein were obtained (Supplementary document Figure S1). This protein was similar to that identified in our previous study [15] and was used throughout the present study to investigate its biological function. There were no significant differences in any of the growth parameters at 29 dpv before the challenge test.

### 3.2. Assessment of Serum IgM Specific to S. agalactiae Using ELISA

Significant differences were observed in the IgM specific to *S. agalactiae* in the WC+CC vaccine group compared with those in both the WC vaccine and control groups at 8 dpv. Furthermore, at 15 dpv, the IgM specific to *S. agalactiae* in the WC vaccine group increased to a level comparable to that in the WC+CC vaccine group, which exhibited higher levels relative to those in the unvaccinated groups (*p* < 0.05). Moreover, at 29 dpv, the WC+CC group exhibited significantly higher levels of *S. agalactiae*-specific IgM compared with both the WC and control groups, with a noticeable decrease observed at 29 dpv (*p* < 0.05).

**Figure 1:**
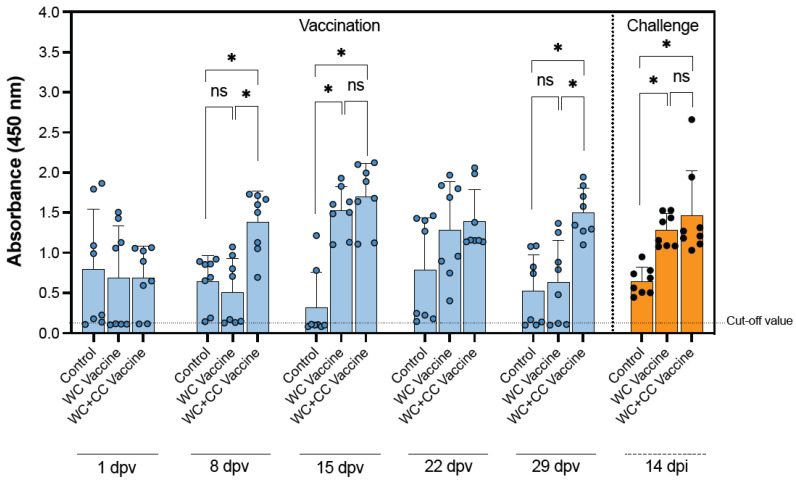
Weekly antibody titers of IgM antibodies specific to *S. agalactiae* in the control and vaccinated fish and fish survival after challenge with *S. agalactiae* 14 days postvaccination (dpv). The data are presented as the mean ± SD. The statistical significance of differences between the control and treatment groups is indicated by * (*p* < 0.05); ns, not significant.

Furthermore, surviving fish challenged with *S. agalactiae* exhibited significantly higher levels of IgM specific to *S. agalactiae* in both the WC and WC+CC vaccine groups than in the control groups. On the other hand, significant differences in specific IgM antibodies in the surviving fish between the WC and WC+CC vaccine groups at 14 dpi were not observed (*p* > 0.05) (Figure 1).

### 3.3. Analysis of the Expression of Immune-Related Genes

**Figure 2:**
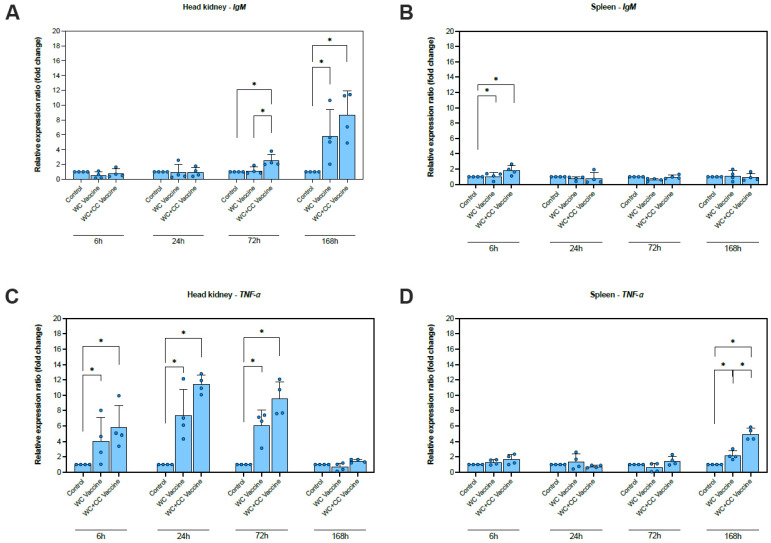

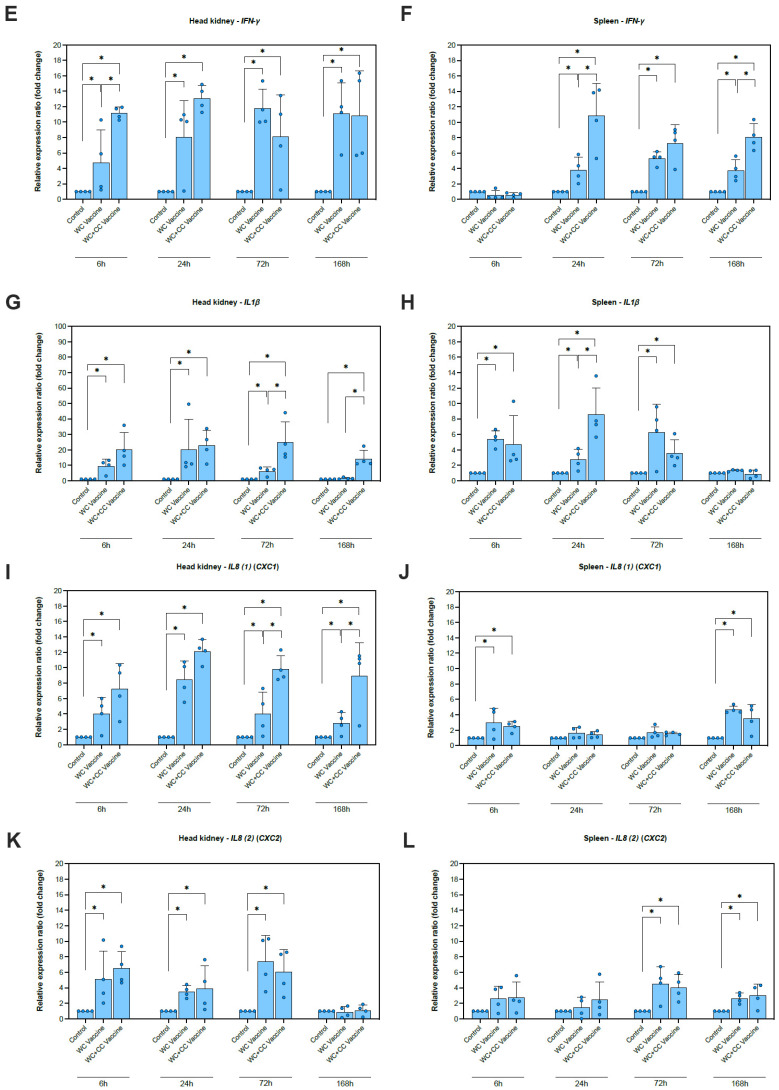

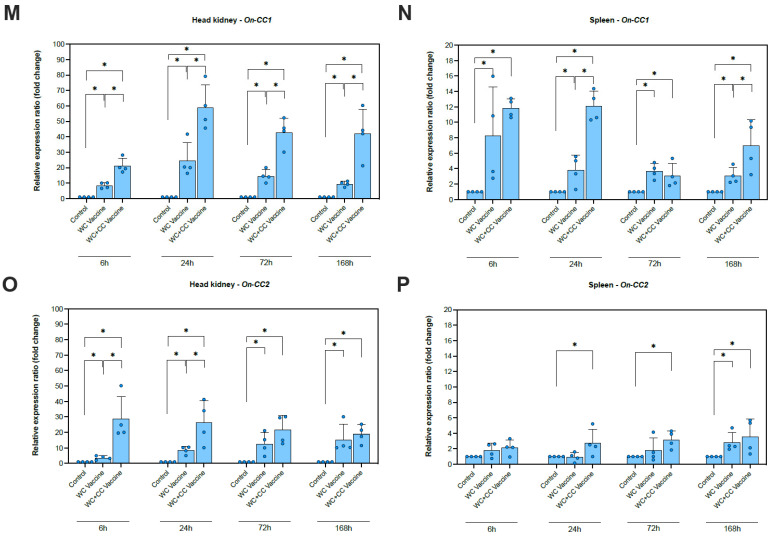
Analysis of the expression of immune-related genes (*IgM*, *TNF-α*, *IFN-γ*, *IL-1β*, *IL-8(1) (CXC1)*, *IL-8(2) (CXC2)*, *On-CC1*, and *On-CC2*) in the head kidney (**A**,**C**,**E**,**G**,**I**,**K**,**M** and **O**, respectively) and spleen (**B**,**D**,**F**,**H**,**J**,**L**,**N** and **P**, respectively) of Nile tilapia. The significant differences between the unvaccinated and vaccinated groups are indicated by * (*p* < 0.05); ns, not significant.

The relative expression levels of eight immune-responsive genes were assessed to evaluate immune responses at the molecular level in the spleen and head kidney at 6, 24, 72, and 168 hpv (Figure 2).

A significantly higher increase in *IgM* expression was detected in the WC+CC vaccine group than in the unvaccinated and WC vaccine groups at 72 hpv. Moreover, both the WC and WC+CC groups exhibited significantly higher expression than the control group, with increases of approximately 6–8-fold (*p* < 0.05). The difference between the WC and WC+CC groups was not significant at 168 hpv (*p* > 0.05) (Figure 2A). Additionally, at only 6 hpv, a significantly higher increase in IgM expression was observed in the spleen in both the WC and WC+CC vaccine groups than in the unvaccinated group, with increases of approximately 1.5–2-fold (*p* < 0.05). Moreover, significant differences in *IgM* expression between the WC and WC+CC groups were not observed. In the spleen, there were no significant differences in the *IgM* expression observed from 24 to 168 hpv among all the test groups (*p* > 0.05) (Figure 2B).

The *TNF-α* gene was clearly more upregulated in the vaccinated group than in the unvaccinated group at 6, 24, and 72 hpv (*p* < 0.05). There were no significant differences in *TNF-α* expression in the kidney between the WC and WC+CC vaccine groups (*p* > 0.05). At 168 hpv, significant differences in *TNF-α* expression in the kidneys among the groups were not observed (*p* > 0.05) (Figure 2C). However, in the spleen, more significant upregulation of *TNF-α* expression was found at 168 hpv. Compared with those in the unvaccinated group, TNF expression in the WC and WC+CC vaccine groups was significantly upregulated, and TNF-α levels in the spleen were significantly higher in the WC+CC group than in the WC-vaccinated group (*p* < 0.05) (Figure 2D).

Compared with the control group, the expression of *IFN-γ*, which is one of the genes examined, in the head kidney increased greatly in both vaccinated groups at 6, 24, 72, and 168 hpv and in the spleen at 24, 72 (Figure 2E), and 168 (*p* < 0.05) hpv (Figure 2F). Notably, the differences in *IFN-γ* expression between the WC and WC+CC groups were significant in the head kidney at 6 hpv and in the spleen at 24 and 168 hpv (*p* < 0.05) (Figure 2F).

Compared with the control group, the expression of *IL-1β* in the spleen and head kidney was highly elevated in both the WC and WC+CC vaccine groups at 6, 24, and 72 hpv. Significant differences in *IL-1β* expression in the head kidney between the WC and WC+CC groups at 72 and 168 hpv were detected (*p* < 0.05). Additionally, in the spleen, significant differences in *IL-1β* expression at 168 hpv were not observed (*p* > 0.05) (Figure 2G,H).

Compared with the control group, the expression of *IL-8(1)* (*CXC1*) in the head kidney at 6, 24, 72, and 168 hpv and in the spleen at 6 and 168 hpv was highly upregulated in both the WC and WC+CC vaccine groups. Notably, significant differences in *IL-8(2)* expression between the WC and WC+CC groups were observed at 72 and 168 hpv in the head kidney (*p* < 0.05) (Figure 2I,J). Moreover, in comparison with the unvaccinated group, the expression of *IL-8(2)* (*CXC2*) in the head kidney at 6, 24, and 72 hpv and in the spleen at 72 and 168 hpv was highly upregulated in the vaccinated groups (*p* < 0.05). No significant differences in *IL-8(2)* transcript levels between the WC and WC+CC groups were detected at any time point in either the head kidney or the spleen (*p* > 0.05) (Figure 2K,L).

Compared with the unvaccinated group, the transcription of *On-CC1* in both the spleen and head kidney was highly upregulated in both the WC and WC+CC vaccine groups at all time points examined (6, 24, 72, and 168 hpv). Significant differences between the WC- and WC+CC-vaccinated groups were observed in the upregulation of *On-CC1* expression in the head kidney at all time points and in the spleen at 24 and 168 hpv (*p* < 0.05) (Figure 2M,N).

Compared with the unvaccinated group, *On*-*CC2* expression was highly upregulated in the head kidney in both the WC and WC+CC vaccine groups at all time points examined (6, 24, 72, and 168 hpv). Significant differences in *On*-*CC2* expression between the WC and WC+CC groups at 6 and 24 hpv were detected in the head kidney (*p* < 0.05). Notably, *On*-*CC2* transcriptional levels in the spleen were significantly higher in the WC+CC vaccine groups than in the WC and unvaccinated groups at 24 and 72 hpv (*p* < 0.05). Interestingly, the WC group also exhibited initial upregulation of *On*-*CC2* at 168 hpv, reaching a level similar to that of the WC+CC group (Figure 2O,P).

### 3.4. Transcriptome Analysis

#### 3.4.1. Transcriptome Sequencing and Annotation

Transcriptome analysis of head kidney tissue was effectively performed using Illumina HiSeq^TM^ at 24 and 96 hpv for both the WC and WC+CC vaccine groups. The libraries were designated as WC_24, WC_96, WC+CC_24, and WC+CC_96. For the nonvaccinated fish (control), sequencing was performed only at 96 hpv after injection with PBS, and the resulting data were then compared with those of both the 24 and 96 hpv samples of groups vaccinated with the WC and WC+CC vaccines in the transcriptome analysis. To ensure data integrity, reads containing adapters and low-quality reads were removed, resulting in an average of 39,894,306–46,654,700 raw reads. Subsequently, this refinement procedure yielded approximately 38,299,708–45,289,016 clean reads for all examined samples. The average Q scores for the clean reads at Q20 and Q30 for all sequences ranged from 96.53% to 97.36% and from 91.05% to 92.91%, respectively. The overall GC content averaged approximately 45.79%–48.08% (Table S1).

#### 3.4.2. Sample Correlation Coefficient and Gene Set Analysis

**Figure 3:**
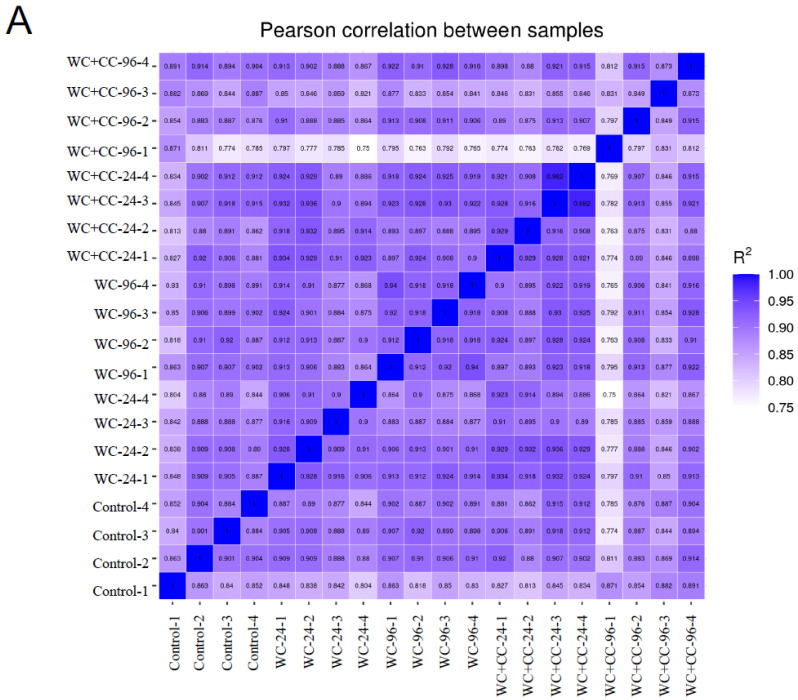

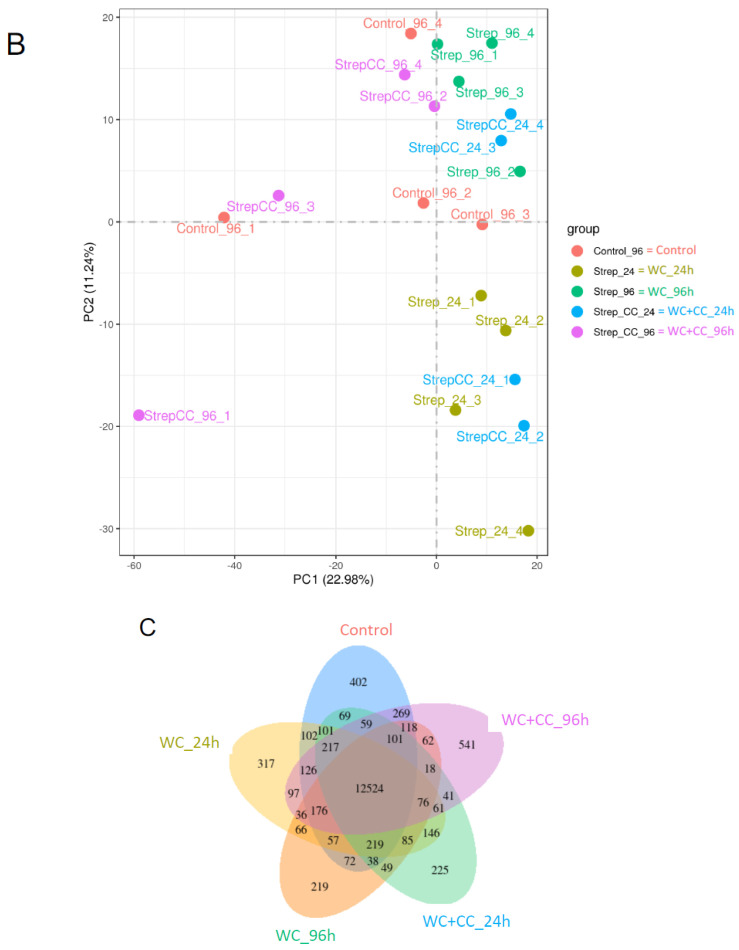
The Pearson correlation coefficient of differences between all samples in the transcriptome analysis (**A**), a scatter plot in two dimensions using a PCA plot of the clustering of sample replicates is shown in (**B**), and gene expression within a group and the overlap representing the genes expressed in common between the groups are expressed by a Venn diagram (**C**).

The Pearson correlation coefficient comparison among samples of each group is shown in Figure 3A. Additionally, the clustering of two-dimensional sample replicates via a principal component analysis (PCA) plot is shown in Figure 3B. Based on the clustered patterns, the WC_24, WC+CC_24, and WC+CC_96 groups exhibited different expression patterns from those of the control and WC_96 groups (Figure 3B). Gene expression within a group and the overlap of genes differentially expressed in common among the groups are shown by a Venn diagram in Figure 3C. This diagram demonstrated that there were 12,524 DEGs shared among all test groups, and 402, 317, 219, 225, and 541 DEGs were specifically expressed in the Control–WC+CC_96 group (Figure 3C).

#### 3.4.3. Analysis of Differentially Expressed Genes (DEGs)

**Figure 4:**
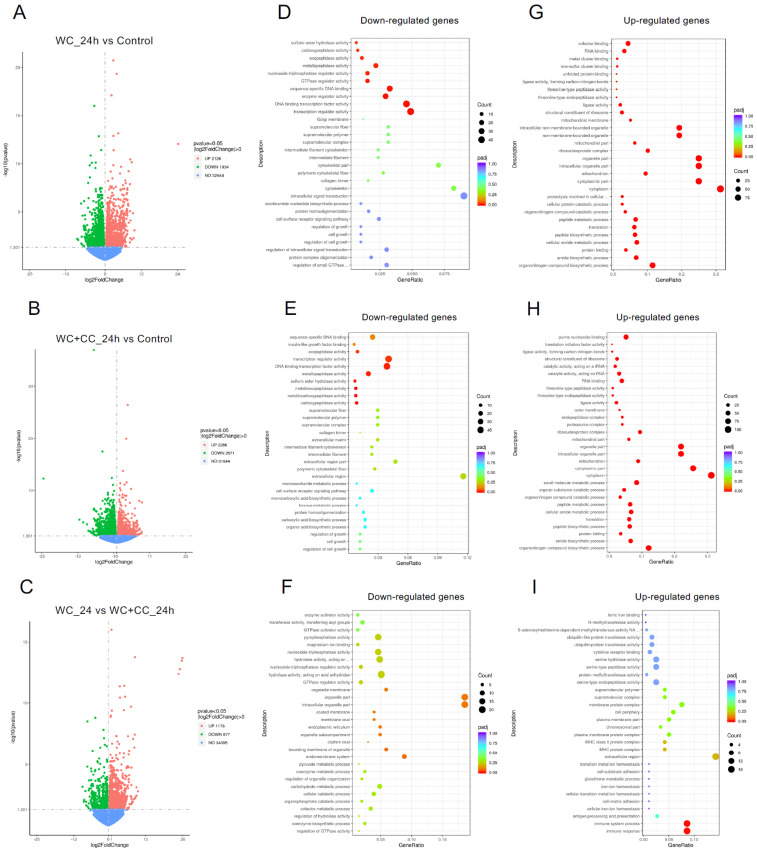
Comparison of DEGs by GO enrichment analysis for both downregulated and upregulated genes at 24 hpv (**A**–**I**). The variation in DEGs was analyzed between the WC and Control (**A**,**D**,**G**), WC+CC and Control (**B**,**E**,**H**), and WC and WC+CC (**C**,**F**,**I**) groups.

DEGs were identified with the criteria of a |log2-fold change| > 1 and a *p*-value < 0.05. At 24 hpv, a comparison of both vaccinated groups to the control groups revealed 4060 DEGs for the WC group vs. the control group, with 2126 upregulated and 1934 downregulated genes (Figure 4A,D,G, respectively). For the WC+CC vs. control comparison, there were 4857 DEGs, with 2286 upregulated and 2571 downregulated genes (Figure 4B,E,H, respectively). Additionally, 2053 DEGs with 1176 upregulated and 877 downregulated genes were found between the WC and WC+CC groups (Figure 4C,F,I, respectively).

**Figure 5:**
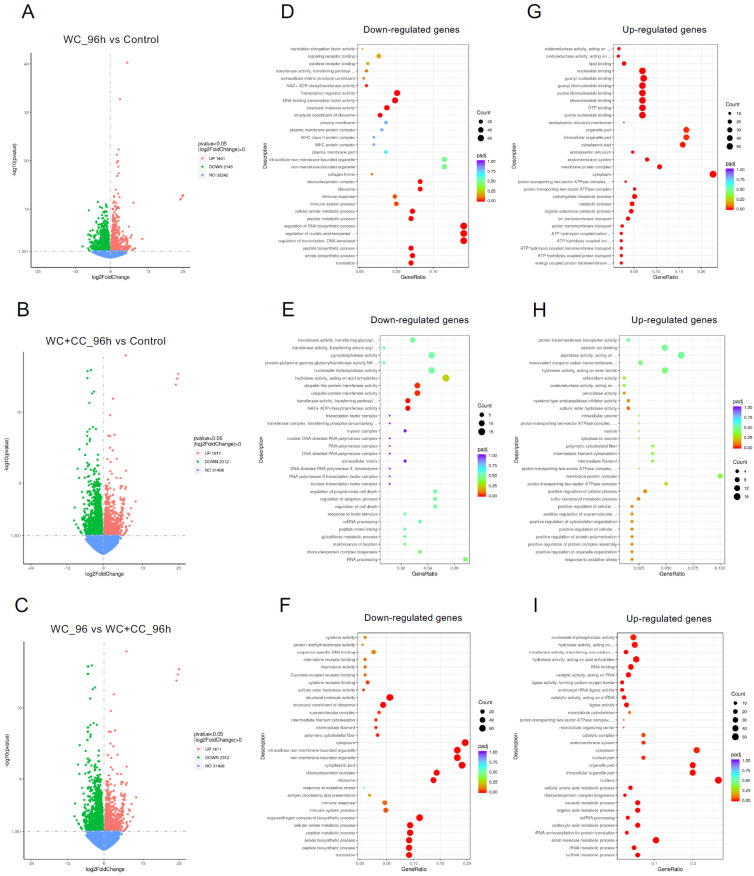
Comparison of DEGs by GO enrichment analysis for both downregulated and upregulated genes at 96 hpv (**A**–**I**). The variation in DEGs was analyzed between the WC vs. Control (**A**,**D**,**G**), WC+CC vs. Control (**B**,**E**,**H**), and WC vs. WC+CC (**C**,**F**,**I**) groups.

At 96 hpv, a comparison of the WC vs. control groups revealed 3746 DEGs, with 1601 and 2145 upregulated and downregulated genes, respectively (Figure 5A,D,G). For the WC+CC vs. control comparison, there were 4857 DEGs, with 2286 and 2571 upregulated and downregulated genes, respectively (Figure 5B,E,H, respectively). Moreover, 3923 DEGs were identified between the WC and WC+CC groups, with 1611 upregulated and 2312 downregulated genes (Figure 5C,F,I, respectively)**.**

#### 3.4.4. GO Enrichment Analysis

**Figure 6:**
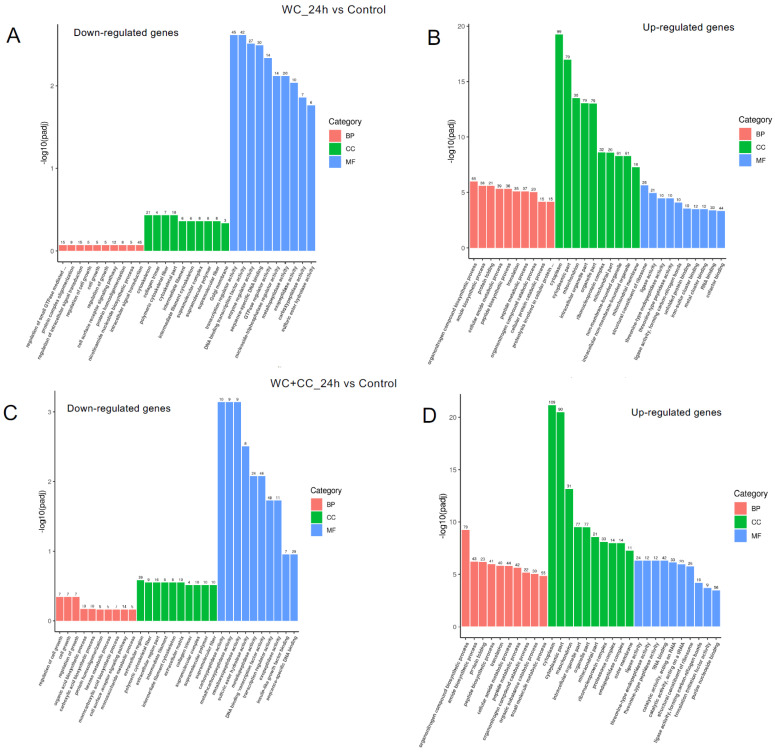

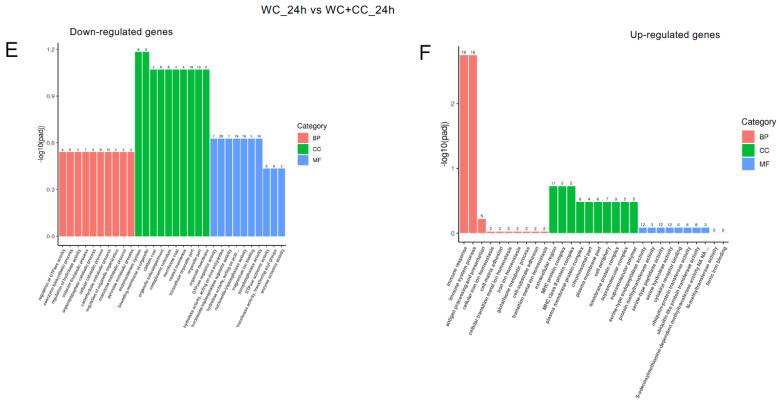
Annotation of all unigenes from all obtained samples annotated according to the three major GO categories, namely, cellular component (CC), biological process (BP), and molecular function (MF), for both downregulated and upregulated DEGs at 24 hpv, comparing the WC vs. Control (**A**,**B**), WC+CC vs. Control (**C**,**D**), and WC vs. WC+CC (**E**,**F**) groups.

The analysis of GO enrichment of DEGs across all observed groups is depicted in Figure 6A–F. At 24 hpv, downregulated genes were significantly enriched primarily in the molecular function (MF) category, followed by the cellular component (CC) and biological process (BP) categories, as observed in both the WC vs. Control and WC+CC vs. Control comparisons. Conversely, the upregulated genes were significantly enriched mainly in the CC category, followed by the BP and MF categories. Moreover, in the comparison between the WC and WC+CC groups at 24 hpv, downregulated genes exhibited significant enrichment primarily in the CC category, followed by the MF and BP categories. Upregulation was predominantly observed in immune responses, immune system processes, and antigen processing and presentation in the BP category, followed by the CC and MF categories.

**Figure 7:**
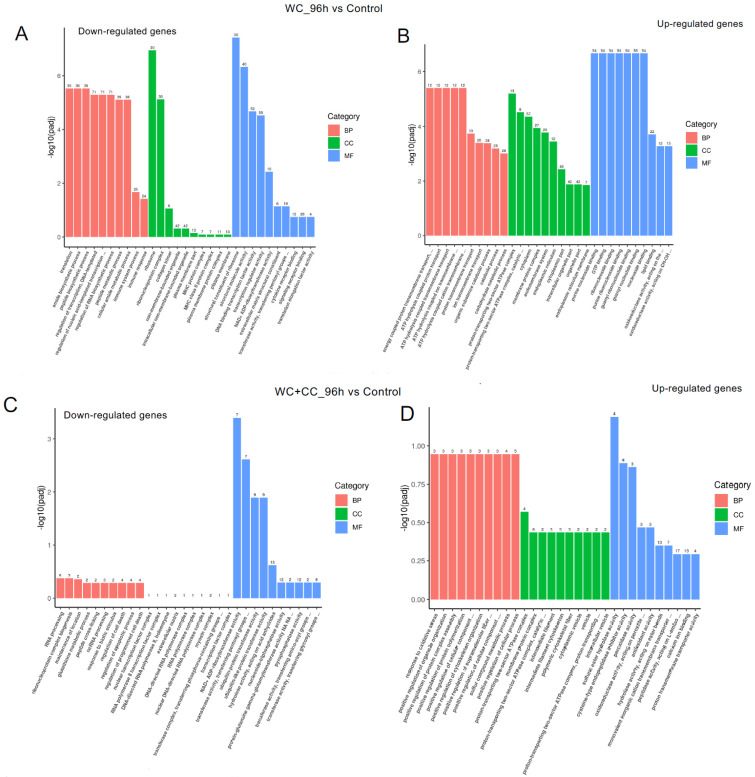

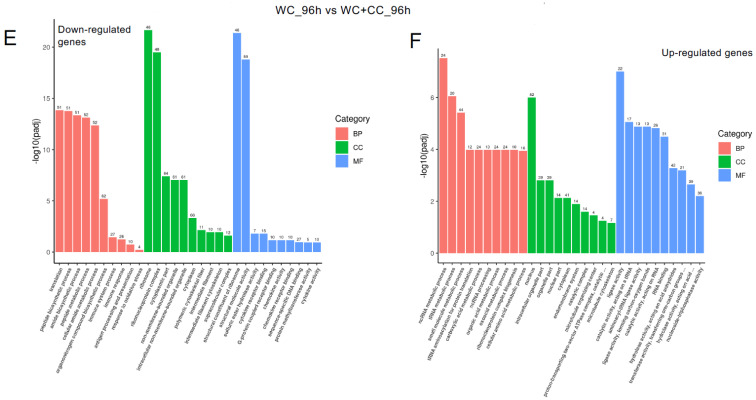
Annotation of all unigenes from all samples annotated according to the three major GO categories, cellular component (CC), biological process (BP), and molecular function (MF), for both downregulated and upregulated DEGs at 96 hpv, comparing the WC vs. Control (**A**,**B**), WC+CC vs. Control (**C**,**D**), and WC vs. WC+CC (**E**,**F**) groups.

At 96 hpv, downregulation was partly observed in all three categories, BP, CC, and MF, but robust upregulation was more pronounced between the WC and control groups (Figure 7A,B). Additionally, more genes related to the CC, BP, and MF categories were upregulated in the WC+CC group than in the control unvaccinated group (Figure 7C,D). Moreover, in the comparison between the WC and WC+CC groups at 96 hpv, downregulated genes exhibited significant enrichment primarily in the partial BP, CC, and MF categories. Upregulation was predominantly observed across all categories (Figure 7A–F).

#### 3.4.5. Analysis of Differential Gene Expression (DGE)

**Figure 8:**
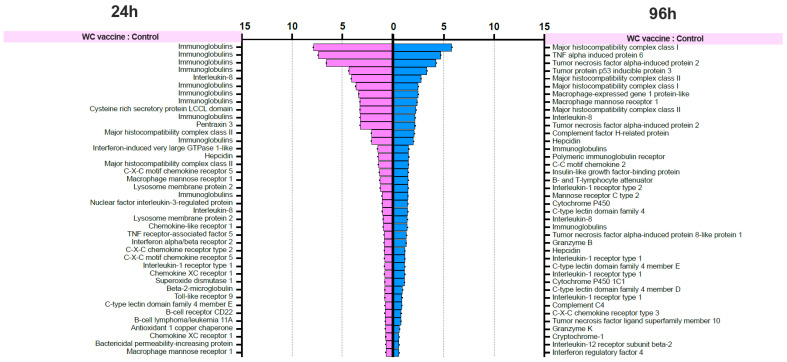

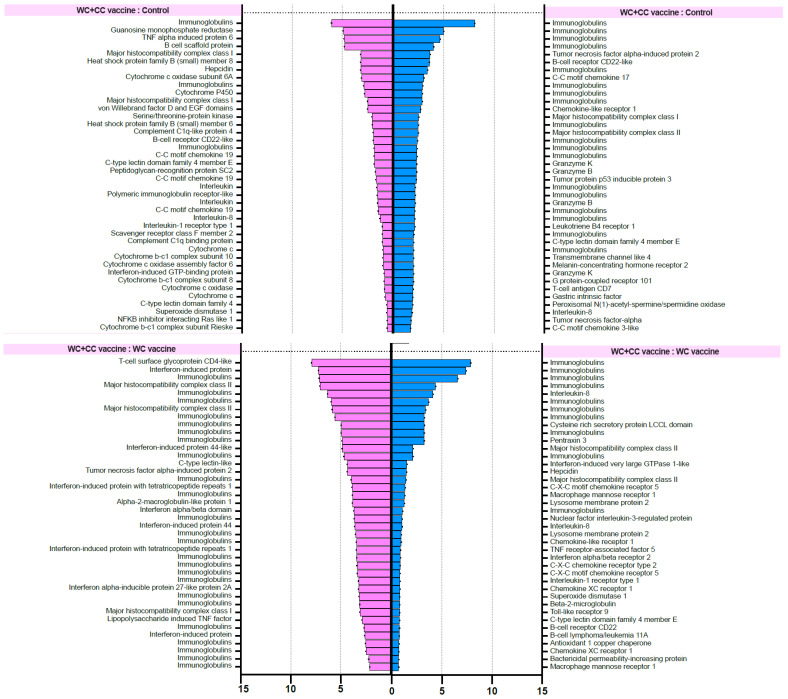
List of forty upregulated immune-related genes in vaccinated Nile tilapia at 24 and 96 hpv in the WC vs. Control, WC+CC vs. Control, and WC vs. WC+CC groups.

The differential gene expression (DGE) analysis data were derived from the FPKM values, and the *p* values were evaluated. We focused on the 40 most upregulated immune-responsive genes across all treatments. Additionally, we analyzed the top 40 upregulated immune-related genes in eukaryotic animals to assess host immune defense mechanisms. The top 40 upregulated immune-related genes are presented in Figure 8.

RNA-seq investigation of immune-responsive gene expression in fish vaccinated with the WC and WC+CC vaccines at 24 hpv revealed the predominant upregulation of several immune-related genes. However, in the comparison analysis between the WC and unvaccinated groups, notably upregulated genes in the WC group included immunoglobulin (13.16%), B-cell receptor CD22-like (13.16%), major histocompatibility complex class I (*MHC I*) (10.53%), cytochrome P450 3A40 (8.55%), and heat shock protein beta-11 (7.02%). Additionally, granzyme K (4.39%), hepcidin (4.39%), and domain family 4 member E of C-type lectin (3.51%) exhibited moderate upregulation. These findings highlight the distinct expression profiles of DEGs between the WC vaccine and unvaccinated groups, with immunoglobulins and the B-cell receptor CD22-like being prominently upregulated (Figure 8).

In contrast, in the WC+CC group vs. the unvaccinated group comparison, immunoglobulins were the most highly upregulated genes (7.32%), as were immune-related genes such as TNF alpha-induced protein 6 (4.88%), B-cell scaffold protein (4.88%), and *MHC I* (3.25%). Notably, interleukin-8, C-C motif chemokine 19, and interleukin also exhibited notable upregulation (each approximately 0.81%). Conversely, genes such as interleukin-1 receptor type 1 and cytochrome c showed minor upregulation, each with a prevalence of approximately 0.81%. These results highlight distinct gene expression patterns between the WC+CC vaccine and control groups, with immunoglobulins being prominently upregulated (Figure 8).

However, in the comparison between the WC and WC+CC vaccine groups at 24 hpv, immunoglobulin was the most notably upregulated gene (47.06%). Other genes, such as interferon-induced protein (14.71%), interferon-induced protein 44 (5.88%), and major histocompatibility complex class II (*MHC II*) (5.88%), were also significantly upregulated. Moreover, several genes, including T-cell surface glycoprotein CD4-like, C-type lectin-like, and alpha-2-macroglobulin-like protein 1, demonstrated lower levels of upregulation, each at approximately 2.94%. Overall, these findings illustrate distinct gene expression patterns between the WC and WC+CC vaccine groups, with immunoglobulin being the most prominently upregulated gene (Figure 8).

On the other hand, in the comparison analysis between the WC and control vaccine groups at 96 hpv, *MHC I* was the most notably upregulated gene (11.76%). Other genes, such as tumor necrosis factor alpha-induced protein 2 (5.88%), TNF alpha-induced protein 6 (5.88%), and tumor protein p53 inducible protein 3 (2.94%), were also significantly upregulated. Moreover, several genes, including macrophage mannose receptor 1, macrophage-expressed gene 1 protein-like, and *MHC II*, were downregulated by approximately 2.94%. Overall, these findings highlight distinct gene expression patterns between the WC and WC+CC vaccine groups, with *MHC I* being the most prominently upregulated gene (Figure 8).

In the comparison analysis between the WC+CC vaccine group and the control group at 96 hpv, immunoglobulin was the most notably upregulated gene (36.84%). Other genes, such as tumor necrosis factor alpha-induced protein 2 (5.26%), B-cell receptor CD22-like (2.63%), and C-C motif chemokine 17 (2.63%), were also significantly upregulated. Moreover, several genes, including *MHC I*, *MHC II*, granzyme K, granzyme B, and tumor protein p53 inducible protein 3, were less upregulated, each at approximately 2.63%. Overall, these findings highlight distinct gene expression patterns between the WC and WC+CC vaccine groups, with immunoglobulin being the most prominently upregulated gene during this specific period (Figure 8).

Moreover, in the comparison between the WC and WC+CC vaccine groups at 96 hpv, immunoglobulin was the most notably upregulated gene (28.21%). Other genes, such as interleukin-8 (7.54%), cysteine-rich secretory protein LCCL domain (3.77%), and pentraxin 3 (3.77%), were also significantly upregulated. Moreover, several genes, including *MHC II*, interferon-induced very large GTPase 1-like, and hepcidin, were less upregulated, at approximately 3.77%. Overall, these findings highlight distinct gene expression patterns between the WC and WC+CC vaccine groups, with immunoglobulin being the most prominently upregulated gene (Figure 8).

### 3.5. Challenge Test

**Figure 9:**
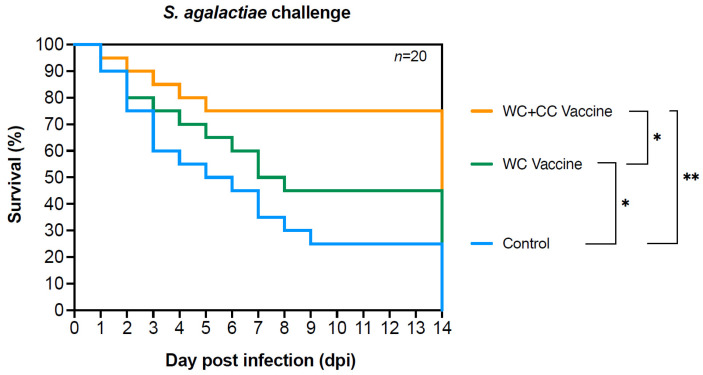
Survival analysis curve showing the trend of fish death after Nile tilapia challenge with *S. agalactiae* in the control fish and fish vaccinated with the WC or the WC+CC formulation. Different asterisks indicate statistically and highly significant differences between groups; * *p* < 0.05 and ** *p* < 0.01, respectively.

Compared with unvaccinated fish, fish vaccinated with either the WC or WC+CC vaccine exhibited significantly higher survival rates after challenge with *S. agalactiae*, with rates of 73.33% and 54.67%, respectively, and an RPS of 64.9 and 40.36%, respectively, which were 24.00%. Additionally, significant differences in survival were recorded between fish vaccinated with the WC and the WC+CC vaccines, with significantly higher survival in the WC+CC vaccine group (Figure 9).

## 4. Discussion

Tilapia farming is critical for Thailand’s economy; however, severe bacterial epidemics, particularly of harmful diseases caused by *S. agalactiae*, have occurred, resulting in substantial economic deprivation [2]. Various practical and safe strategies have been investigated to address these challenges, including the administration of probiotics, immunostimulants from various organisms, and vaccines [33,34]. Among these methods, vaccination against *S. agalactiae* infection has been the most beneficial [35], but the enhancement of vaccine efficacy by means of high-potential adjuvants, which are produced by the Nile tilapia immune system itself, is very restricted.

To date, five different chemokine subfamilies have been identified in mammals and teleosts. These chemokines include the CX3C and XC chemokines, which are absent in teleosts; CXC and CC chemokines, which are found in both mammals and teleosts; and a teleost-specific CX chemokine [14]. For CC chemokines in fish, seven subgroups of CC chemokines, namely, the CCL17/22 clade, CCL20 clade, CCL19/21/25 clade, CCL27/28 clade, monocyte chemotactic protein (MCP) clade, macrophage inflammatory protein (MIP) clade, and a fish-specific CC clade, have been identified based on their phylogenetic relationships [13]. This study focused on the CC chemokine protein (*On*-CC1), which is grouped into a conserved fish-specific CC chemokine clade and recognized for its essential function in the vertebrate immune system [13,14]. The protein acts as a bridge between innate and acquired immunity, aiding in pathogen elimination [13,14,15]. By elucidating the immune mechanisms of CC chemokines, this research aimed to inform strategies for enhancing tilapia immunity against streptococcosis and offer valuable insights for mitigating bacterial outbreaks in aquaculture.

In the present study, we evaluated immunological responses and assessed the enhancement of the potency of the *S. agalactiae* vaccine using a recombinant CC chemokine (r*On*-CC1) as an adjuvant. Efficient r*On*-CC1 production was achieved using *E. coli*, providing a cost-effective production system. Minimal amounts of this protein (0.2 µg) effectively stimulate the immune system in tilapia, with no observed adverse effects even at higher doses (100 µg), indicating its safety for in vivo use [15].

The assessment of serum IgM concentrations specific for *S. agalactiae* by ELISA revealed insights into the immunogenicity of the WC+CC vaccine compared to that of the WC vaccine alone. Our results demonstrated significant differences in the concentrations of specific *S. agalactiae* IgM between the WC+CC vaccine group and both the WC vaccine and control groups at 8 dpv. This result suggested that the addition of CC protein as an adjuvant enhances the immune response, leading to heightened levels of specific IgM earlier after vaccination. Furthermore, at 15 dpv, while the levels of specific IgM in the WC vaccine group increased to a level comparable to those observed in the WC+CC vaccine group, the latter group exhibited higher levels relative to those in the control groups, indicating prolonged and greater immune activation with the WC+CC vaccine. Interestingly, at 29 dpv, the WC+CC group maintained significantly higher levels of *S. agalactiae*-specific IgM than both the WC vaccine and control groups, although with a noticeable decrease, suggesting a potential weakening of the immune response over time [36]. Moreover, our results revealed that surviving fish challenged with *S. agalactiae* exhibited significantly higher amounts of *S. agalactiae*-specific IgM in both the WC- and WC+CC-vaccinated groups than in the unvaccinated group, indicating the effectiveness of both vaccines in eliciting an immune response against *S. agalactiae*. However, significant differences between the WC and WC+CC vaccine groups at 14 dpi were not observed, suggesting that the two vaccine formulations had comparable protective effects against *S. agalactiae* challenge. Overall, these results highlight the potential of the WC+CC vaccine as an effective adjuvant formulation for enhancing the immune response against *S. agalactiae*, leading to sustained and superior antibody levels compared to those of the WC vaccine alone [16,17,18,19].

The comparison of immune-related gene expression profiles between fish vaccinated with the WC and the WC+CC vaccines at various time points postvaccination revealed the adjuvant effect of *On*-CC1 in enhancing vaccine-induced immune responses. Our results indicate that both the WC and WC+CC vaccines elicited a significant increase in immune-responsive genes compared to the control group, underscoring their effectiveness in stimulating immune activation. However, notable differences in the expression patterns of immune-related genes emerged between the two vaccine formulations, suggesting that the *On*-CC1 adjuvant had a distinct adjuvant effect. Notably, the upregulation of *IgM* in the WC+CC vaccinated group was much higher than that in both the WC vaccine and unvaccinated groups at 72 hpv, indicating that a potentiated immune response was facilitated by the presence of *On*-CC1 as an adjuvant [37,38], similar to that in other fish vaccines, including CCL4, which significantly increased *IgM* levels and disease resistance against infection of cyprinid herpesvirus 2 (CyHV-2) in gibel carp (*Carassius auratus gibelio*) [39]; CCL19 and CCL4, which are potent adjuvant compounds for application in the development of VAA DNA vaccines against *Vibrio anguillarum* infection [19]; and rRbCC1, which exhibited adjuvant properties and clear synergism for an injected vaccine in rock bream (*Oplegnathus fasciatus*) against *Streptococcus iniae* infection [40].

Although both the WC and WC+CC groups exhibited substantially higher *IgM* levels than the control group, the addition of *On*-CC1 further augmented this response, as evidenced by the significant difference observed between the WC+CC and WC vaccine groups at 72 hpv. Interestingly, while the significant difference between the WC- and WC+CC-vaccinated groups was not significant at 168 hpv in the kidney, the WC+CC group demonstrated more significant upregulation than the WC group in the spleen, suggesting differential immune responses induced by the two vaccine formulations [40].

Recently, various cytokines, especially inflammatory cytokines [41], which strongly induce immune responses during and after vaccine immunization, have been intensively investigated as key immunogenicity regulators in various fish species [42] and are worth investigating in fish vaccine trials.

In the present study, *TNF-α* gene expression was markedly higher in the WC- and WC+CC-vaccinated groups than in the unvaccinated group at multiple time points, indicating that a robust inflammatory response was elicited by both the WC and WC+CC vaccines [43,44]. While no significant differences were detected between the WC and WC+CC vaccine groups in the kidney, more significant differences were detected at 168 hpv in the spleen, with the WC+CC group exhibiting higher upregulation of *TNF-α* expression than the WC group. Similarly, *IFN-α* was highly upregulated in all vaccinated groups compared to the unvaccinated group, with significant differences between the WC and WC+CC groups observed in the head kidney at 6 hpv and in the spleen at 24 hpv, further highlighting the enhanced immune activation conferred by the WC+CC vaccine [45,46]. Moreover, *IL-8(1)* and (*CXC1*) *IL-1β* transcriptional levels were markedly increased in both the WC and WC+CC vaccine groups compared to those in the control unvaccinated group at multiple time points, with significant differences between the WC and WC+CC vaccinated groups observed at specific time points. Generally, TNF-α and IL-1β strongly increase the transcriptional levels of CCL28 in air-exposed epithelial cells, which are specifically driven by an NFκB-dependent pathway [47], and IL-8 or a CXC chemokine that strongly drives CC chemokine responses to immunocapture remote monocytes to reach inflammatory sites [48], suggesting that *On*-CC1 also induces the transcription of both TNF-α and IL-1β in the Nile tilapia immune system. Additionally, *On-CC1* and *On*-CC2 exhibited highly upregulated transcriptional levels in both vaccinated groups compared to those in the unvaccinated group, with significant differences between the WC and WC+CC groups found at various time points in both the spleen and head kidney. Notably, the WC+CC vaccine group showed significantly higher upregulation of *On*-CC2 expression in the spleen at 24 and 72 hpv compared to the WC and control groups, suggesting a sustained adjuvant effect of the *On*-CC1 protein that efficiently induced the transcriptional levels of *On*-*CC1* itself and *On*-*CC2* following vaccine administration. In addition to chemotaxis effects, similar to IL-2, various CC chemokines can strongly induce the activation and proliferation of natural killer cells (NK cells), and all CC chemokines are capable of inducing CC chemokine-activated killer (CHAK) responses upon incubation with CD56+ cells, suggesting that the primary effectors are natural NK cells [49], which are crucial players in the cellular innate immune system. The induction of immune responses via vaccination with *On*-CC1 as an adjuvant may induce more robust responses, resulting in increased vaccine efficacy. Overall, these results underscore the potential of the WC+CC vaccine as an effective adjuvant formulation for augmenting immune-related gene expression and enhancing immune responses compared to those of the WC vaccine alone.

To date, transcriptome analysis has been commonly employed to investigate immune responses during various pathogenic infections and vaccination trials in several fish species and even in Nile tilapia [28,50,51,52]. However, the adjuvant effects of *On*-CC1 during *S. agalactiae* vaccination of Nile tilapia are still unknown. To better understand the adjuvant properties of *On*-CC1 during inactivated *S. agalactiae* vaccination of Nile tilapia, RNA-seq was applied. In this study, transcriptomic investigation revealed significant upregulation of several immune-related genes, such as immunoglobulins, B-cell receptor CD22-like, MHC I, cytochrome P450 3A40, and heat shock protein beta-11, at the mRNA level in the WC vaccine group. These findings underscore the ability of the WC vaccine to stimulate a robust immune defense mechanism, although certain gene expression profiles predominated. Notably, immunoglobulins and the B-cell receptor CD22-like were prominently upregulated, indicating their critical roles in facilitating humoral immune responses and antigen recognition, respectively. This finding differed from the transcriptional profiles of the gills of Nile tilapia injected with live *S. agalactiae*, in which various genes related to immune response pathways, such as the nucleotide oligomerization domain (NOD)-like receptor signaling pathway, the Toll-like receptor signaling pathway, the intestinal immune network for immunoglobulin A (IgA), and the cytosolic-DNA sensing pathway, were the predominant significantly upregulated genes [50]. Our findings also differed from those reported by Cui et al. [53], who reported transcriptomic expression profiles in the intestines of Nile tilapia coinfected with *S. agalactiae* and *S. iniae*; C-X-C motif chemokine 10-like (CXCL 10), E3 ubiquitin-protein ligase TRIM39-like, interleukin-1 beta-like, proteasome subunit beta type-8 (*PSMB8*), partial, IgG Fc-binding protein, IgM heavy chain VH region, ATP synthase F(0) complex subunit B1 and C-C motif chemokine 19-like were the predominantly expressed genes.

Conversely, in the WC+CC vaccine group, immunoglobulin remained the most upregulated gene, suggesting a sustained adjuvant effect of *On*-CC1 in potentiating antibody production. Additionally, immune-related genes such as TNF alpha-induced protein 6, B-cell scaffold protein, and major histocompatibility complex class I exhibited notable upregulation, further indicating the enhancement of immune activation by the inclusion of *On*-CC1 as an effective adjuvant in the formulation. Interestingly, upregulated expression of various interleukins and chemokines was also observed, indicating the involvement of inflammatory and chemotactic pathways in the immune response elicited by the WC+CC vaccine [43,44,45,46,47,48,49], supporting findings from previous gene expression analysis experiments using qRT–PCR.

Moreover, the comparison between the WC and WC+CC vaccine groups revealed significant differences in gene expression profiles, with immunoglobulin being the most prominently upregulated gene in the WC+CC group across multiple time points. This result suggested that the addition of *On*-CC1, a powerful adjuvant, amplifies the vaccine-induced humoral immune response, leading to increased antibody production [36,37,38,39,40]. Furthermore, the upregulation of other immune-related genes, such as interferon-induced proteins and *MHC II* genes, underscores the broader enhancement of immune activation conferred by the WC+CC vaccine than by the WC vaccine alone. These different findings not only indicate the adjuvant effects of *On*-CC1 during Nile tilapia vaccination but also reveal that other factors, such as organ, fish age, and pathogen form (inactivated (vaccines) or live), should be further investigated.

Finally, the results of the challenge test demonstrated the efficacy of both the WC and WC+CC vaccines in conferring protection against a harmful *S. agalactiae* infection, as evidenced by significantly higher survival rates in the WC and WC+CC groups compared to the control group. Fish vaccinated with either formulation exhibited substantial increases in survival rates, with the WC and WC+CC vaccines resulting in survival rates of 54.67% and 73.33%, respectively, compared to a survival rate of only 24.00% in the control group. These findings underscore the important role of vaccination in enhancing the ability of fish to combat bacterial infections and mitigate disease-related mortality.

Furthermore, the comparison between the WC and WC+CC vaccine groups revealed notable differences in survival outcomes, with the WC+CC vaccine group exhibiting a significantly higher survival rate than the WC vaccine group. This observation suggested that the inclusion of the adjuvant *On*-CC1 in the vaccine formulation leads to enhanced protective immunity against *S. agalactiae* challenge. The significant increase in survival rates observed in the WC+CC vaccine group highlights the adjuvant effect of *On*-CC1 in augmenting vaccine-induced immune responses, thereby improving the ability of the fish to tolerate pathogen exposure and reduce mortality.

## 5. Conclusions

Our findings highlight the potent adjuvant effect of CC chemokines in augmenting immune-related gene expression and enhancing the efficacy of the WC vaccine in stimulating immune defense mechanisms. The distinct gene expression patterns observed between the WC and WC+CC vaccine groups in the transcriptome analysis underscore the important roles of adjuvants in modulating the immune systems of fish to vaccination, ultimately contributing to improved vaccine efficacy and host protection against pathogenic *S. agalactiae*. The significant differences in survival after pathogenic bacterial challenge between the WC and WC+CC vaccine groups emphasize the importance of adjuvants in optimizing vaccine-induced immune responses and maximizing host resistance to bacterial pathogens. Thus, the use of the WC+CC vaccine holds promise for enhancing disease control measures in aquaculture settings, ultimately contributing to the sustainability and productivity of fish farming operations.

## Data Availability

The data supporting this study’s findings are available upon request from the corresponding authors.

## Supplementary Materials

The following supporting information can be downloaded at https://www.mdpi.com/article/10.3390/vaccines12060641/s1. Figure S1: Overexpression of r*On*-CC1 in the *E. coli* system, Table S1: Summary of RNA-seq data quality for each sample.
