# Supplementary material for: Adjuvant Effects of a CC Chemokine for Enhancing the Efficacy of an Inactivated Streptococcus agalactiae Vaccine in Nile Tilapia (Oreochromis niloticus)
Source: Vaccines (Basel). 2024 Jun 8;12(6):641. doi: 10.3390/vaccines12060641 (PMC11209360; doi:10.3390/vaccines12060641)
Supplement: Supplementary file 1 [file vaccines-12-00641-s001.zip › vaccines-2995015-supplementary.pdf]

Supplementary Document Figure S1: Overexpression of r*On*-CC1 in *E. coli* system.

Purified recombinant protein CC1

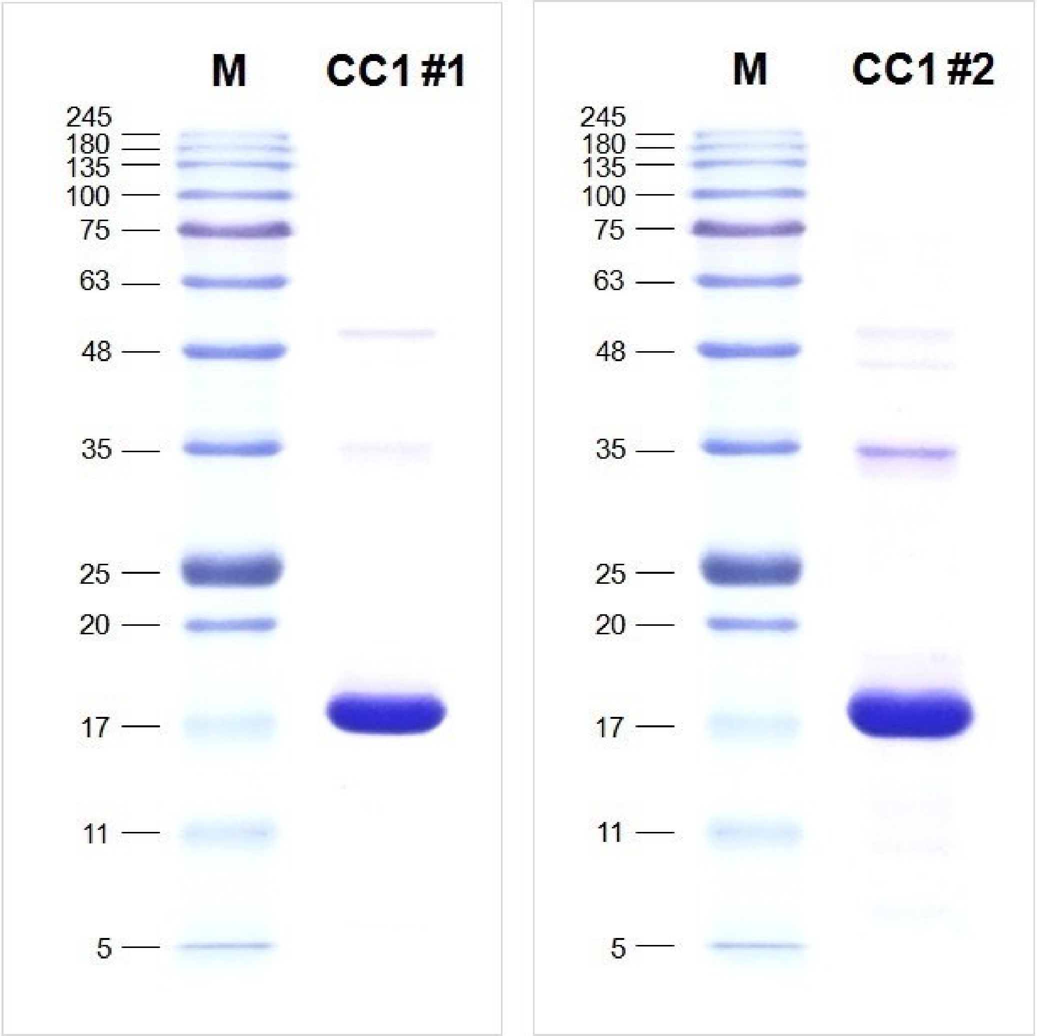

Purified recombinant protein	Conc. (µg/µl)	Volume (µl)	Total protein (mg)
CC1 #1	14.3	1000µl x 5 tubes	71.5
CC1 #2	45.9	700µl x 1 tube	32.1
**Total**			**103.6**

CC1 #1 = 5 µg/well

CC1 #2 = 5 µg/well

Supplementary Document Table S1. Summary of RNA-seq data quality for each sample.

Sample	Library	Raw_reads	Raw_bases	Clean_reads	Clean_bases	Error_rate	Q20	Q30	GC_pct
Control-1	FRRB220295112-1r	43,076,836	6.46G	41,613,178	6.24G	0.03	97.08	92.28	46.66
Control-2	FRRB220295113-1r	42,917,046	6.44G	41,560,320	6.23G	0.03	97.33	92.76	47.43
Control-3	FRRB220295114-1r	44,121,236	6.62G	42,599,384	6.39G	0.03	97.13	92.39	47.92
Control-4	FRRB220295115-1r	46,654,700	7.00G	45,289,016	6.79G	0.03	96.53	91.05	47.92
WC-24-1	FRRB220295116-1r	43,928,912	6.59G	42,542,068	6.38G	0.03	97.26	92.67	47.11
WC-24-2	FRRB220295117-1r	43,019,122	6.45G	41,654,460	6.25G	0.03	97.14	92.37	47.57
WC-24-3	FRRB220295118-1r	43,216,444	6.48G	41,719,246	6.26G	0.03	97.25	92.62	47.36
WC-24-4	FRRB220295119-1r	42,756,950	6.41G	41,277,352	6.19G	0.03	97.31	92.73	47.61
WC-96-1	FRRB220295120-1r	43,240,972	6.49G	41,565,892	6.23G	0.03	97.35	92.91	47.09
WC-96-2	FRRB220295121-1r	43,273,264	6.49G	42,106,684	6.32G	0.03	97.36	92.88	48.08
WC-96-3	FRRB220295122-1r	42,663,458	6.40G	41,381,592	6.21G	0.03	97.29	92.67	46.87
WC-96-4	FRRB220295123-1r	42,253,476	6.34G	41,045,942	6.16G	0.03	97.19	92.51	47.20
WC+CC-24-1	FRRB220295124-1r	42,831,194	6.42G	41,649,914	6.25G	0.03	97.12	92.29	47.39
WC+CC-24-2	FRRB220295125-1r	45,303,516	6.80G	44,075,148	6.61G	0.03	97.06	92.18	46.89
WC+CC-24-3	FRRB220295126-1r	42,780,452	6.42G	41,319,672	6.20G	0.03	97.03	92.11	47.68
WC+CC-24-4	FRRB220295127-1r	45,048,840	6.76G	43,861,508	6.58G	0.03	96.96	92.01	47.91
WC+CC-96-1	FRRB220295128-1r	39,894,306	5.98G	38,299,708	5.74G	0.03	97.31	92.81	45.79
WC+CC-96-2	FRRB220295129-1r	44,139,438	6.62G	42,738,398	6.41G	0.03	97.31	92.65	46.21
WC+CC-96-3	FRRB220295130-1r	42,872,662	6.43G	41,831,798	6.27G	0.03	97.15	92.36	46.86
WC+CC-96-4	FRRB220295131-1r	43,424,926	6.51G	42,070,362	6.31G	0.03	97.22	92.54	47.18
